# Supplementary material for: Distinct mechanisms of replication stress induced by oncogenic RAS and cyclin E1 converge on R-loop-dependent fork reversal
Source: Nat Commun. 2026 Apr 3;17:4784. doi: 10.1038/s41467-026-71353-8 (PMC13219726; doi:10.1038/s41467-026-71353-8)
Supplement: Supplementary file 1 — Supplementary Information [file 41467_2026_71353_MOESM1_ESM.pdf]

Distinct mechanisms of replication stress induced by oncogenic RAS and cyclin E1 converge on R-loop-dependent fork reversal

Anna Oravetzova, Marketa Dvorakova, Anca-Irina Mihai, Martin Andrs, Margarita Sobol, Anton Zuev, Kaustubh Shukla, Barbora Boleslavska, Vinicio Rosano, Christiane König, Jiri Prokes, Hana Hanzlikova, Libor Macurek, Jana Dobrovolna and Pavel Janscak

Supplementary Information

Supplementary Figures 1-14

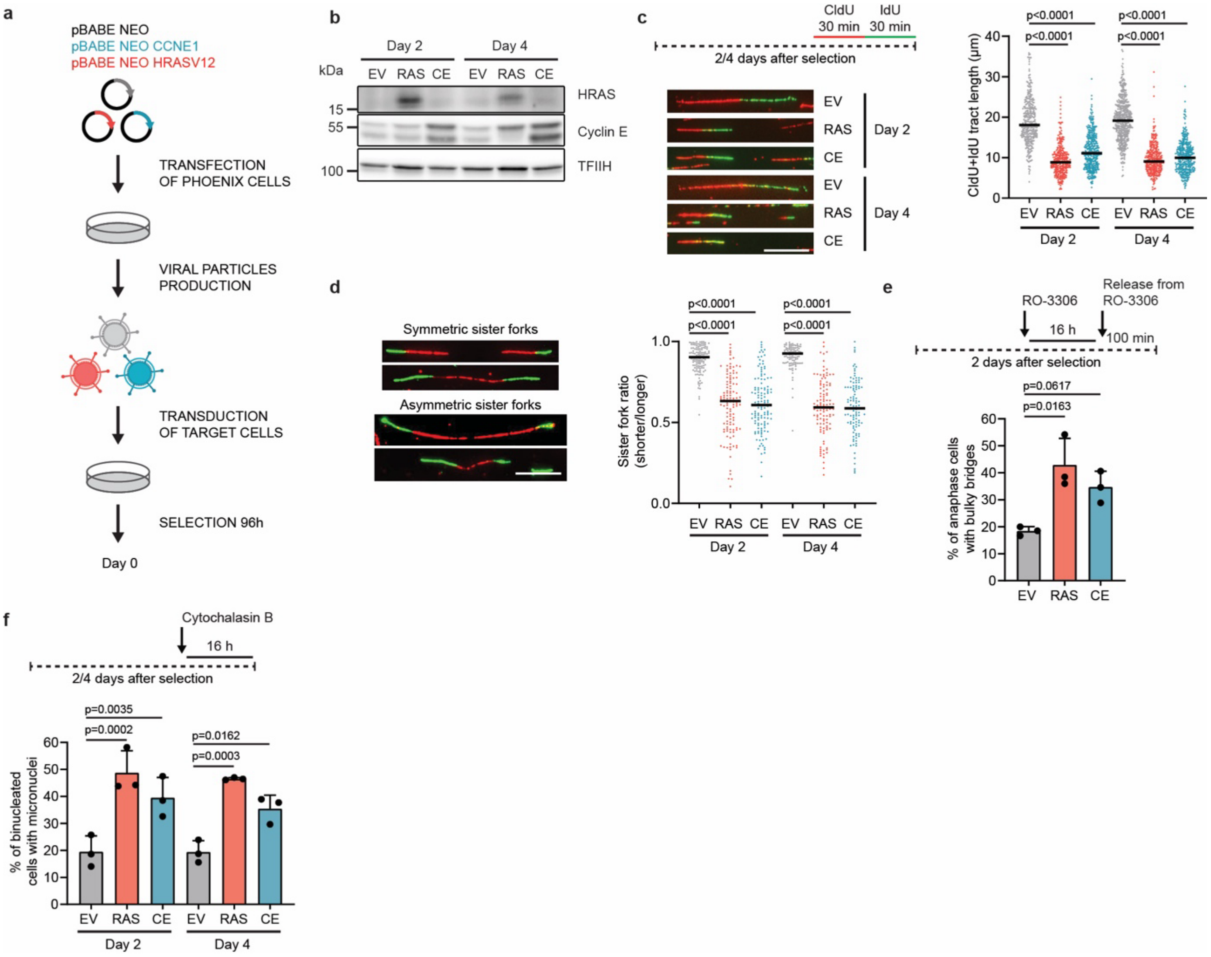

Supplementary Fig. 1. Ectopic overexpression of HRASV12 or cyclin E1 induces DNA replication stress and chromosome mis-segregation in mitosis.

a Workflow for retroviral transduction and selection. Phoenix-AMPHO cells were transfected with pBABEneo derivatives encoding for HRASV12 (RAS) or cyclin E1 (CE), or with empty vector (EV). The medium containing viral particles was collected 48, 52 and 72 h post-transfection, respectively, and then incubated with target cells for 3 h in each case. 18 h after the last transduction, cells were selected in G418-containing medium. After approximately 4 days, non-transduced control died (Day 0). Phenotypic analyses were usually performed 2-4 days after non-transduced control died. **b** Western blot analysis of HRASV12 and cyclin E1 expression in transduced U2OS cells 2 or 4 days after selection. **c** *Top-left*: Workflow for DNA fiber labeling in transduced U2OS cells 2 or 4 days after selection. *Bottom-left*: Representative images of replication tracks. Scale bar, 10 µm. *Right:* Plot of values of replication tract lengths (CldU+IdU) from three independent experiments ($n \geq 310$). **d** *Left:* Representative images of symmetric and asymmetric replication tracts of sister forks for DNA fibers in (**c**). Scale bar, 10 µm. *Right:* Plot of values of IdU tract length ratio of sister forks (sister fork ratio) from experiments in (**c**) ($n \geq 101$). **e** *Top*: Workflow for enrichment of anaphase cells. Transduced U2OS cells were treated with 9 µM CDK1 inhibitor RO-3306 for 16 h, then released for 100 min into fresh medium. *Bottom*: Quantification of cells with bulky anaphase bridges. At least 25 anaphase cells were analyzed per experiment for each condition. **f** *Top*: Workflow for enrichment of once-divided binucleated cells by inhibition of cytokinesis. Transduced U2OS cells were incubated with cytochalasin B (2 µg/ml) for 16 h, followed by fixation and DAPI staining. *Bottom*: Quantification of binucleated cells with micronuclei. At least 100 binucleated

cells were analyzed per experiment for each condition. **c**, **d** Black horizontal lines indicate the median; p-values were calculated by Kruskal-Wallis test followed by Dunn's multiple comparisons test. **e**, **f** Data are presented as mean ± SD, n = 3; p-values were calculated by one-way ANOVA followed by Tukey's multiple comparisons test. Source data are provided as a Source Data file.

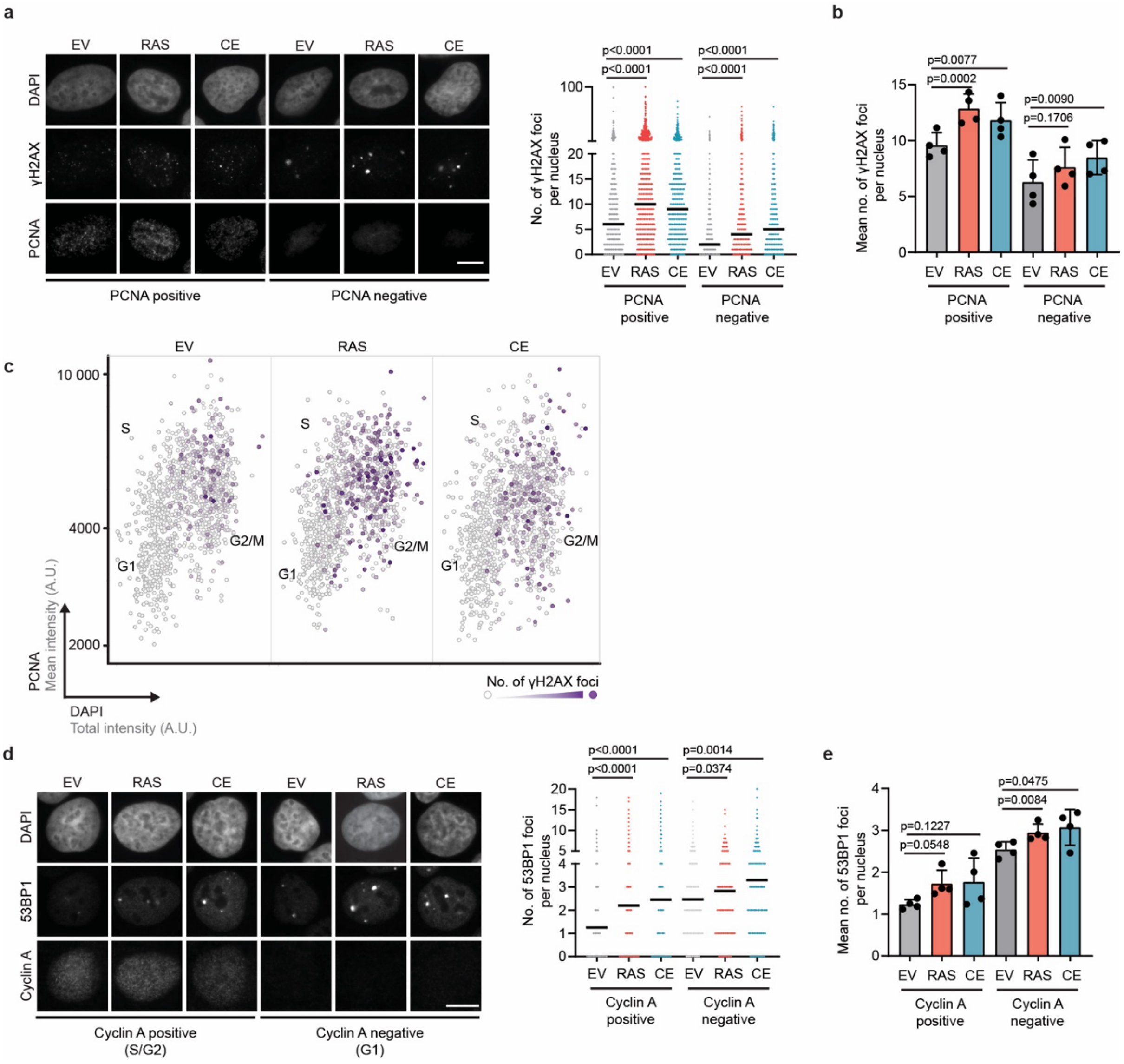

Supplementary Fig. 2. Effect of HRASV12 and cyclin E1 overexpression on the formation of γH2AX and 53BP1 foci in U2OS cells.

a *Left:* Representative images of γH2AX foci in PCNA-positive and PCNA-negative U2OS cells two days after selection. EV, empty vector; RAS, HRASV12; CE, cyclin E1. Scale bar, 10 µm. *Right*: Quantification of the number of γH2AX foci per nucleus in PCNA-positive and PCNA-negative cells ($n \geq 985$). A representative plot from four independent experiments yielding similar results is shown. Black horizontal lines indicate the mean; p-values were calculated by Kruskal-Wallis test followed by Dunn's multiple comparisons test. **b** Plot of the mean values of the data sets represented in (**a**). Data are presented as mean ± SD, n = 4; p-values were calculated by one-way ANOVA followed by Tukey's multiple comparisons test. **c** Scatter plot of total DAPI (x-axis) and mean PCNA (y-axis) intensities in individual cells represented in (**a**). Colors indicate the number of foci, as shown in the legend on the right. For visualization, 985 cells per condition were randomly selected. Clusters of G1, S and G2/M phase cells are marked in the plots. **d** *Left:* Representative images of 53BP1 foci in cyclin A-positive (S/G2) and cyclin A-negative (G1) U2OS cells two days after selection. Scale bar, 10 µm. *Right*: Quantification of the number of 53BP1 foci per nucleus in cyclin A-positive and cyclin A-negative cells ($n \geq 399$). A representative plot from four independent experiments

yielding similar results is shown. Black horizontal lines indicate the mean; p-values were calculated by Kruskal-Wallis test followed by Dunn's multiple comparisons test. **e** Plot of the mean values of the data sets represented in (**d**). Data are presented as mean ± SD, n = 4; p-values were calculated by two-tailed paired t-test. Source data are provided as a Source Data file.

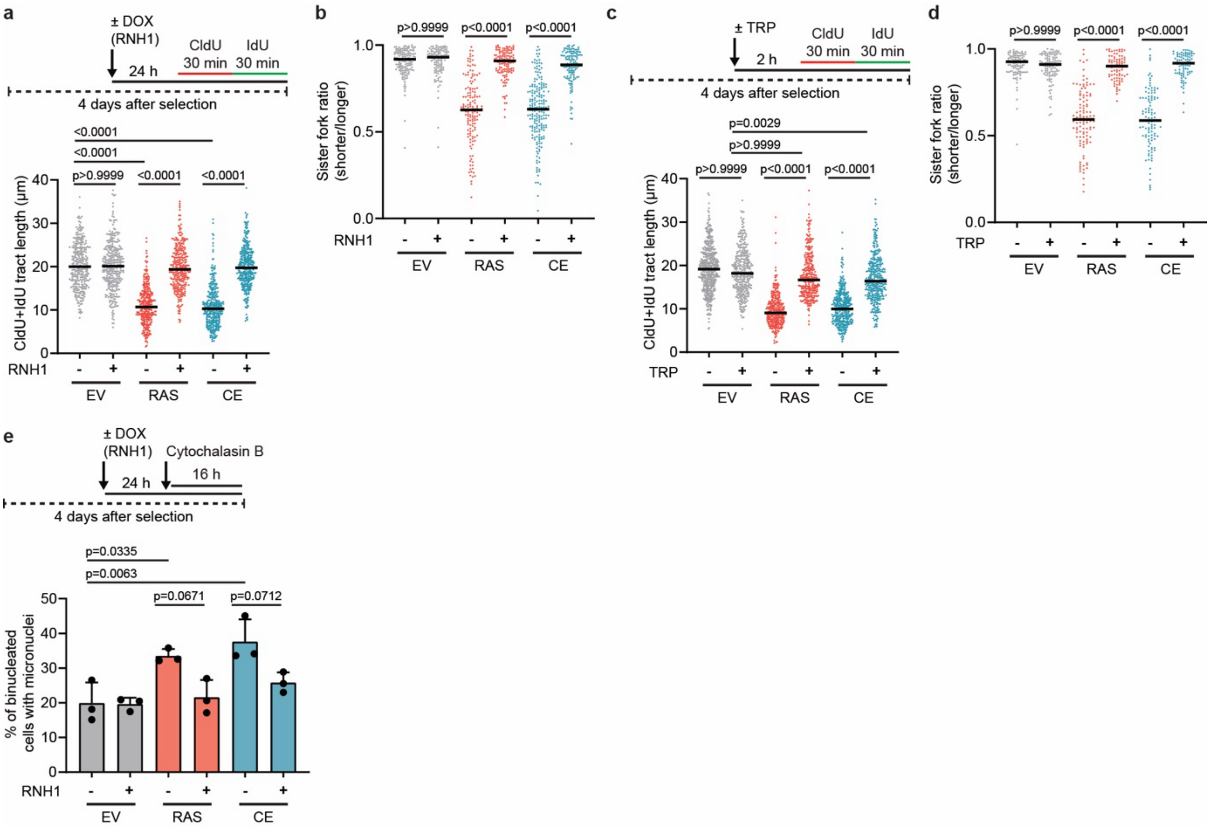

Supplementary Fig. 3. HRASV12- and cyclin E1-induced replication stress is mainly caused by R-loops.

a *Top*: Workflow for DNA fiber labeling in transduced U2OS T-REx [RNaseH1- GFP] cells four days after selection. Where required, cells were treated with doxycycline (DOX; 1 ng/ml) for 24 h to induce RNaseH1-GFP (RNH1) overexpression. *Bottom:* Plot of values of replication tract lengths (CldU+IdU) from three independent experiments ($n \geq 302$). EV, empty vector; RAS, HRASV12; CE, cyclin E1. **b** Plot of values of IdU tract length ratio of sister forks (sister fork ratio) for DNA fibers in (**a**) ($n \geq 113$). **c** *Top:* Workflow for DNA fiber labeling in transduced U2OS cells four days after selection. Where required, the transcription inhibitor triptolide (TRP, 1 μM) was added 1 h before the labeling and was also present during the labeling. *Bottom:* Plot of values of replication tract lengths (CldU+IdU) from three independent experiments ($n \geq 310$). **d** Plot of values of sister fork ratio for DNA fibers in (**c**) ($n \geq 80$). **a-d** Black horizontal lines indicate the median; p-values were calculated by Kruskal-Wallis test followed by Dunn's multiple comparisons test. **e** *Top:* Workflow for enrichment of once-divided binucleated cells by inhibition of cytokinesis. Transduced U2OS T-REx [RNaseH1-GFP] cells were incubated with cytochalasin B (2 μg/ml) for 16 h. Where required, cells were treated with doxycycline (DOX; 1 ng/ml) for 24 h to induce RNase H1-GFP overexpression. Cells were analyzed 4 days after selection. *Bottom:* Quantification of binucleated cells with micronuclei. Data are presented as mean ± SD, n = 3; p-values were calculated by one-way ANOVA followed by Tukey's multiple comparisons test. For each condition, at least 100 binucleated cells were examined for the presence of micronuclei in each experiment. Source data are provided as a Source Data file.

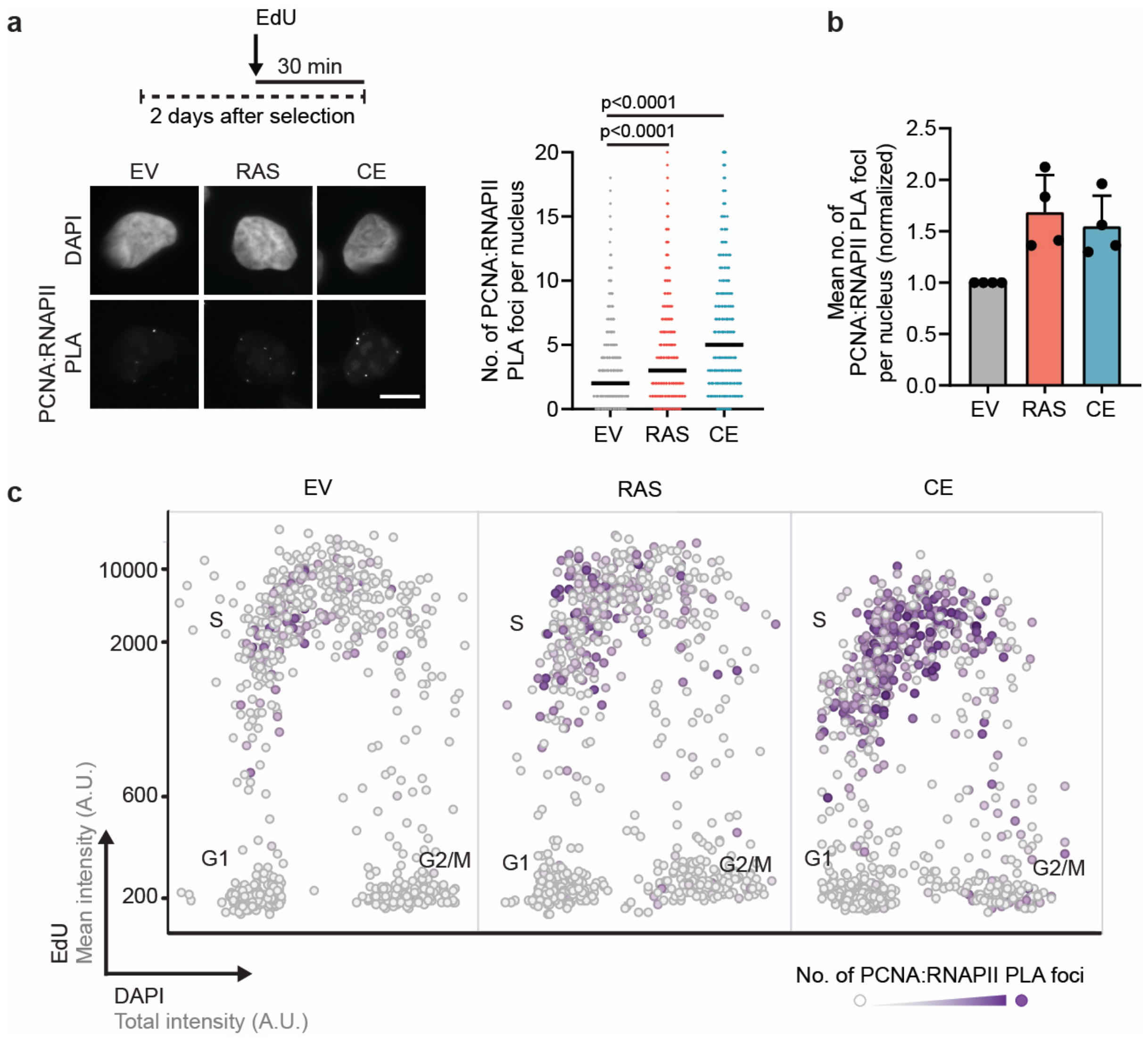

Supplementary Fig. 4. Overexpression of HRASV12 or cyclin E1 increases the frequency of head-on transcription-replication conflicts in U2OS cells.

a *Top-left*: Experimental workflow. 2 days after selection, transduced U2OS cells were pulse-labeled with 25 µM EdU for 30 min and proximity ligation assay (PLA) between PCNA and elongating form of RNA polymerase II (RNAPII) was performed. EV, empty vector; RAS, HRASV12; CE, cyclin E1. *Bottom-left*: Representative images of DAPI and PCNA:RNAPII PLA channels. Scale bar, 10 µm. *Right*: Quantification of the number of PCNA:RNAPII PLA foci for the indicated conditions ($n \geq 775$). A representative plot from four independent experiments yielding similar results is shown. Black horizontal lines indicate the median; p-values were calculated by Kruskal-Wallis test followed by Dunn's multiple comparisons test. **b** Plot of the mean values of the data sets represented in (**a**). Data are normalized to EV. Error bars represent SD, $n = 4$. **c** Scatter plot of total DAPI (x-axis) and mean EdU (y-axis) intensities in individual cells represented in (**a**). Colors indicate the number of PCNA:RNAPII PLA foci, as shown in the legend on the right. For visualization, 775 cells per condition were randomly selected. Clusters of G1, S and G2/M phase cells are marked in the plots. Source data are provided as a Source Data file.

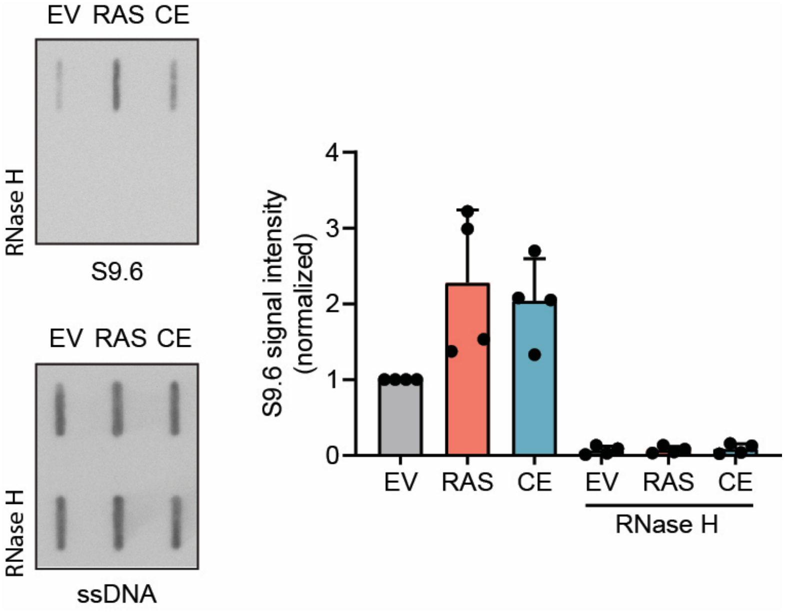

Supplementary Fig. 5. Overexpression of HRASV12 or cyclin E1 induces R-loop formation in U2OS cells.
Slot blot analysis of genomic DNA isolated from transduced U2OS cells on day 2 after selection. Genomic DNA was treated with or without RNase H. Blots were immunostained with S9.6 antibody to detect RNA:DNA hybrids, and with anti-ssDNA antibody for loading control. *Left*: Representative slot blot images. EV, empty vector; RAS, HRASV12; CE, cyclin E1. *Right*: Quantification of S9.6 signal intensity. For each sample, S9.6 signal intensity was normalized to ssDNA intensity. The resulting values are normalized to the EV condition. Error bars represent SD, n = 4. Source data are provided as a Source Data file.

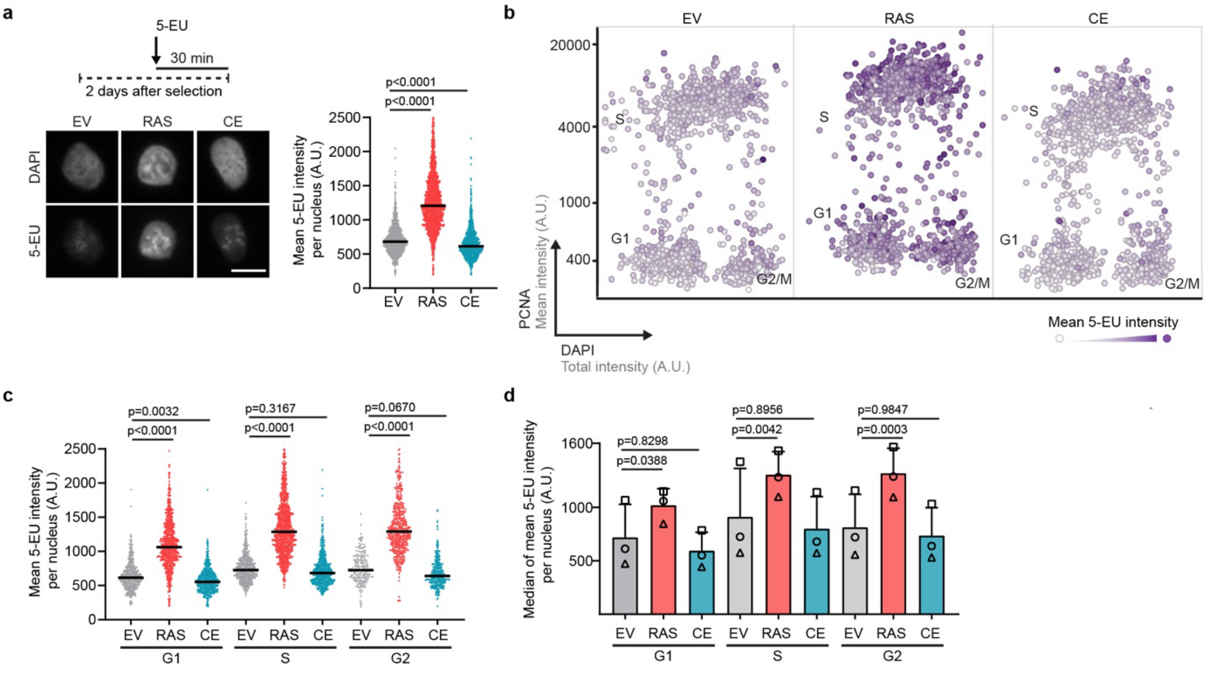

Supplementary Fig. 6. Overexpression of HRASV12 increases transcription in a cell cycle-independent manner.

a *Top-left*: Workflow for nascent transcript labeling by 5-ethynyl uridine (5-EU) incorporation. 2 days after selection, transduced U2OS cells were pulse-labeled with 1 mM 5-EU for 30 min. *Bottom-left*: Representative images of DAPI and 5-EU channels. Scale bar, 10 µm. *Right*: Quantification of mean 5-EU intensity in nuclei of cells transduced with HRASV12 (RAS), cyclin E1 (CE) and empty vector (EV), respectively. A representative plot from three independent experiments yielding similar results is shown ($n \geq 1300$). **b** Scatter plot of total DAPI (x-axis) and mean PCNA (y-axis) intensities in individual cells represented in (**a**). Colors indicate the mean 5-EU intensity, as shown in the legend on the right. For visualization purposes, at least 1000 cells per condition were randomly chosen. Clusters of G1, S and G2/M phase cells are marked in the plots. A.U., arbitrary units. **c** Plot of mean 5-EU intensity in nuclei of cells in (**a**), sorted by individual cell cycle phases based on DAPI and PCNA signals. A representative plot from three independent experiments yielding similar results is shown. **a**, **c** Black horizontal lines indicate the median; p-values were calculated by Kruskal-Wallis test followed by Dunn's multiple comparisons test. **d** Plot of the median values from datasets represented in (**a**) and (**c**). Different symbols represent values obtained for individual experiments. Data are presented as mean ± SD, $n = 3$; p-values were calculated by one-way ANOVA followed by Tukey's multiple comparisons test. Source data are provided as a Source Data file.

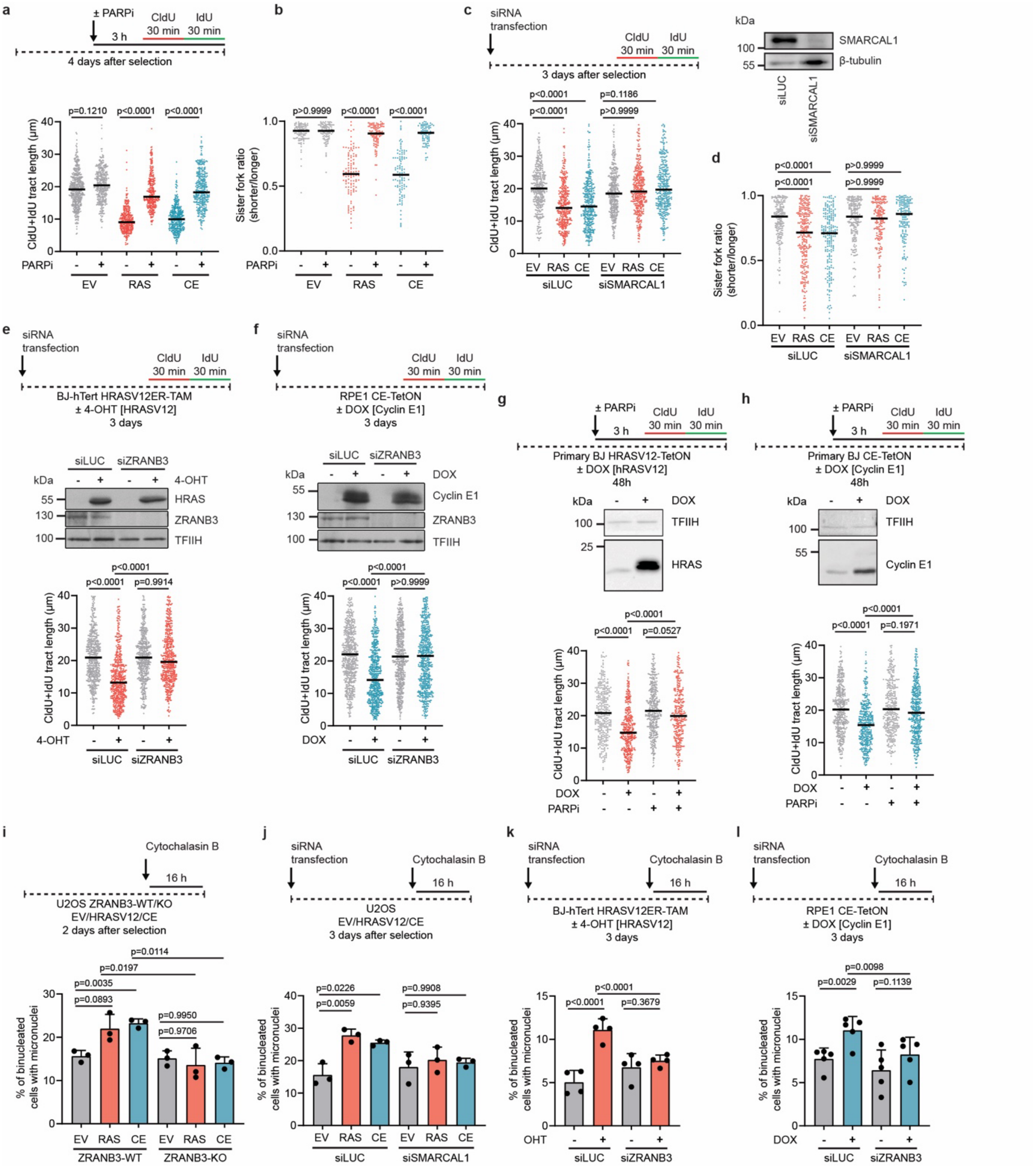

Supplementary Fig. 7. Suppression of replication fork reversal induces unrestrained DNA synthesis and prevents chromosome mis-segregation in cells overexpressing HRASV12 or cyclin E1.

a *Top:* Workflow for DNA fiber labeling in transduced U2OS cells 4 days after selection. PARP1 inhibitor olaparib (PARPi, 10 μM) was present for 2 h prior to and during the labeling. *Bottom:* Plot of values of replication tract lengths (CldU+IdU) from three independent experiments (n ≥ 296). EV, empty vector; RAS, HRASV12; CE, cyclin E1. **b** Plot of the values of sister fork ratio for (**a**) (n ≥ 80). **c** *Top-left:* Workflow for DNA fiber labeling in transduced U2OS cells transfected with with siLUC or siSMARCAL1. *Top-right:* Western blot analysis of SMARCAL1 expression levels. *Bottom-left:* Plot of values of replication tract lengths (CldU+IdU) from three independent experiments (n ≥ 348). **d** Plot of values of sister fork ratio

for (**c**) (n ≥ 170). **e** *Top:* Workflow for DNA fiber labeling in BJ-hTert HRASV12ER-TAM fibroblasts transfected with siLUC or siZRANB3. Cells were treated with 333 nM 4-hydroxytamoxifen (4-OHT) for 72 h to induce HRASV12 expression. *Middle:* Western blot analysis of HRASV12 and ZRANB3 expression levels. *Bottom:* Plot of values of replication tract lengths (CldU+IdU) from four independent experiments (n ≥ 510). **f** *Top:* Workflow for DNA fiber labeling in RPE1 CE-TetON cells transfected with siLUC or siZRANB3. Cells were treated with doxycycline (DOX; 1 μg/ml) for 72 h to induce overexpression of cyclin E1. *Middle:* Western blot analysis of cyclin E1 and ZRANB3 expression levels. *Bottom:* Plot of values of replication tract lengths (CldU+IdU) from four independent experiments (n ≥ 579). **g** *Top:* Workflow for DNA fiber labeling in primary BJ HRASV12-TetON fibroblasts. Cells were treated with DOX (2 μg/ml) for 48 h to induce HRASV12 expression. PARPi was added as in (**a**). *Middle:* Western blot analysis of HRASV12 expression levels. *Bottom*: Plot of values of replication tract lengths (CldU+IdU) from three independent experiments (n ≥ 309). **h** *Top:* Workflow for DNA fiber labeling in primary BJ CE-TetON fibroblasts. Cells were treated with DOX (2 μg/ml) for 48 h to induce cyclin E1 overexpression. PARPi was added as in (**a**). *Middle:* Western blot analysis of cyclin E1 expression levels. *Bottom*: Plot of values of replication tract lengths (CldU+IdU) from three independent experiments (n ≥ 343). **i** *Top:* Workflow for micronucleus assays with transduced U2OS ZRANB3-WT and U2OS ZRANB3-KO cells. Cytochalasin B (2 μg/ml) was added for the last 16 h to enrich for once-divided binucleated cells. *Bottom:* Quantification of binucleated cells with micronuclei. **j** *Top:* Workflow for micronucleus assays with transduced U2OS cells transfected with siLUC or siSMARCAL1. *Bottom*: Quantification of binucleated cells with micronuclei. **k** *Top*: Workflow for micronucleus assays with BJ-hTert HRASV12ER-TAM cells transfected with siLUC or siZRANB3. HRASV12 expression was induced as in (**e**). *Bottom:* Quantification of binucleated cells with micronuclei. **l** *Top:* Workflow for micronucleus assays with RPE1 CE-TetON cells transfected with siLUC or siZRANB3. Cyclin E1 overexpression was induced as in (**f**). *Bottom:* Quantification of binucleated cells with micronuclei. **a-h** Black horizontal lines indicate the median; p-values were calculated by Kruskal-Wallis test followed by Dunn's multiple comparisons test. **i-l** Data are presented as mean ± SD, n = 3-5; p-values were calculated by one-way ANOVA followed by Tukey's multiple comparisons test. At least 100 binucleated cells were analyzed per experiment for each condition. Source data are provided as a Source Data file.

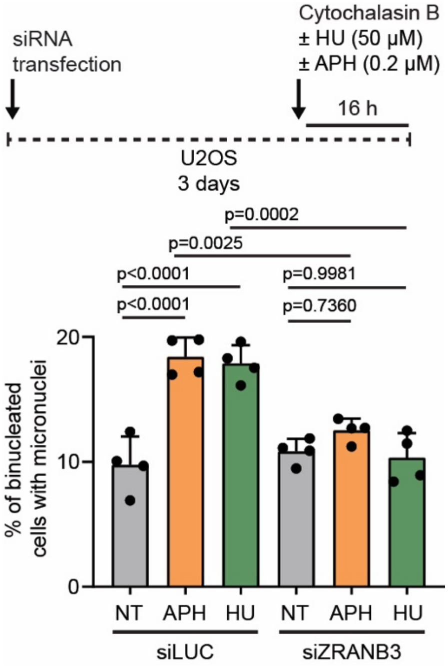

Supplementary Fig. 8. Suppression of replication fork reversal prevents micronucleation induced by low doses of aphidicolin or hydroxyurea.
Top: Workflow for micronucleus assays with U2OS cells. Cells were transfected with siLUC or siZRANB3 and cultured for 72 h. Cytochalasin B (2 µg/ml) was added for the last 16 h to enrich for once-divided binucleated cells. Where indicated, aphidicolin (APH, 0.2 µM) or hydroxyurea (HU, 50 µM) were added together with cytochalasin B. *Bottom:* Quantification of binucleated cells with micronuclei. Data are presented as mean ± SD, n = 4. p-values were calculated by one-way ANOVA followed by Tukey's multiple comparisons test. At least 100 binucleated cells were analyzed per experiment for each condition. Source data are provided as a Source Data file.

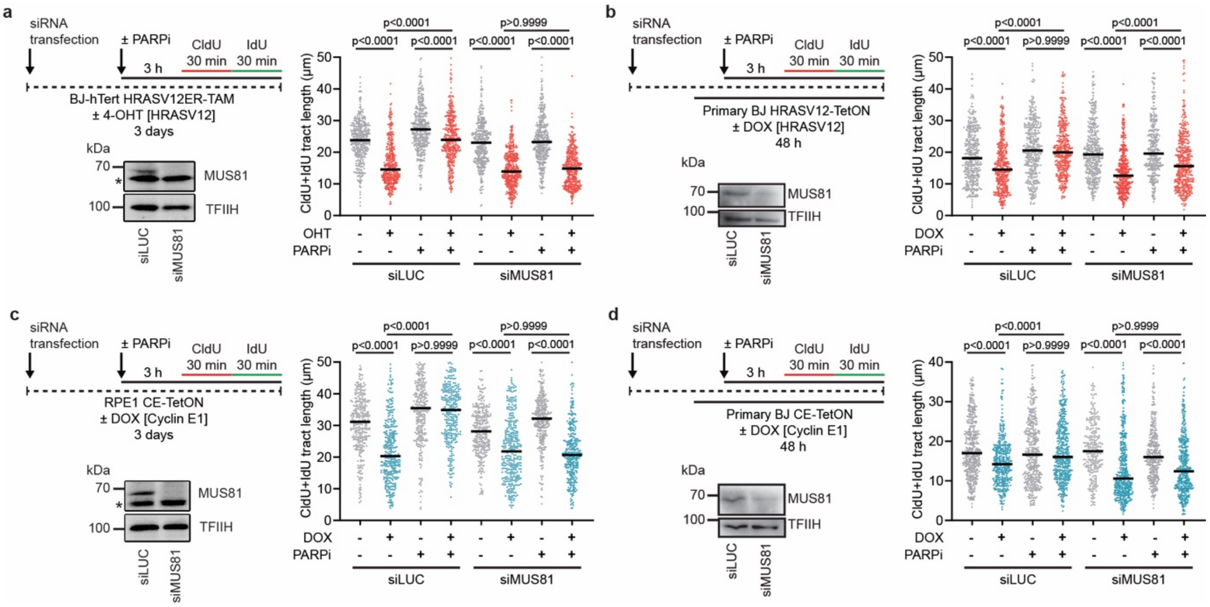

Supplementary Fig. 9. Unrestrained replication fork progression in HRASV12- and cyclin E1-overexpressing cells depends on proteins involved in restarting of R-loop-stalled forks.

a *Top left:* Workflow for DNA fiber labeling in BJ-hTert HRASV12ER-TAM cells transfected with siLUC or siMUS81. Cells were treated with 333 nM 4-hydroxytamoxifen (4-OHT) for 72 h to induce HRASV12 overexpression. PARP inhibitor olaparib (PARPi; 10 µM) was present for 2 h prior to and during the labeling. *Bottom-left:* Western blot analysis of MUS81 expression levels. *Right:* Plot of values of replication tract lengths (CldU+IdU) from three independent experiments ($n \geq 356$). **b** *Top left:* Workflow for DNA fiber labeling in primary BJ HRASV12-TetON fibroblasts transfected with siLUC or siMUS81. Cells were treated with doxycycline (DOX, 2 µg/ml) for 48 h to induce HRASV12 overexpression. PARPi was added as in (**a**). *Bottom-left:* Western blot analysis of MUS81 expression levels. *Right:* Plot of values of replication tract lengths (CldU+IdU) from three independent experiments ($n \geq 335$). **c** *Top left:* Workflow for DNA fiber labeling in RPE1 CE-TetON cells transfected with siLUC or siMUS81. Cell were treated with doxycycline (DOX, 1 µg/ml) for 72 h to induce cyclin E1 overexpression. PARPi was added as in (**a**). *Bottom left:* Western blot analysis of MUS81 expression levels. *Right:* Plot of values of replication tract lengths (CldU+IdU) from three independent experiments ($n \geq 305$). **d** *Top left:* Workflow for DNA fiber labeling in primary BJ CE-TetON fibroblasts transfected with siLUC or MUS81. Cells were treated with DOX (2 µg/ml) for 48 h to induce cyclin E1 overexpression. PARPi was added as in (**a**). *Bottom-left:* Western blot analysis of MUS81 expression levels. *Right:* Plot of values of replication tract lengths (CldU+IdU) from three independent experiments ($n \geq 269$). **a-d** Black horizontal lines indicate the median; p-values were calculated by Kruskal-Wallis test followed by Dunn's multiple comparisons test. The asterisk indicates non-specific band on the MUS81 blots. Source data are provided as a Source Data file.

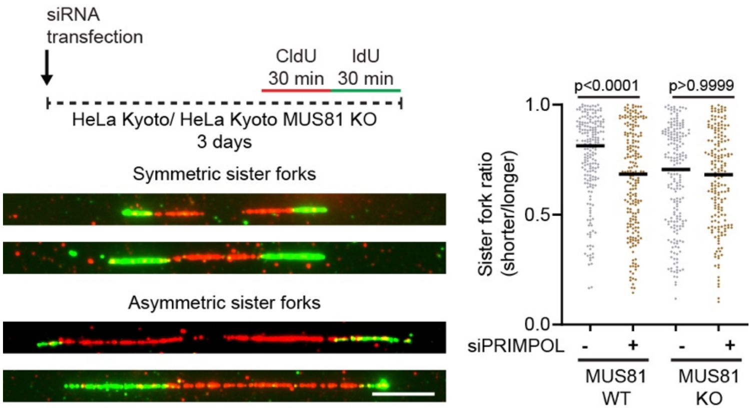

Supplementary Fig. 10. **PRIMPOL and MUS81 act in a common pathway to suppress replication fork stalling.**

Top-left: Workflow for DNA fiber labeling in wild-type (WT) and MUS81 knockout (KO) HeLa Kyoto cells. Cells were transfected with siLUC or siPRIMPOL three days before the labeling. *Bottom-left:* Representative images of symmetric and asymmetric replication tracts of sister replication forks observed on DNA fibers from MUS81 WT and MUS81 KO cells, respectively. Scale bar, 10 µm. *Right:* Plot of values of IdU tract length ratio of sister forks (sister fork ratio) from three independent experiments ($n \geq 185$). Black horizontal lines indicate the median; p-values were calculated by Kruskal-Wallis test followed by Dunn's multiple comparisons test. Source data are provided as a Source Data file.

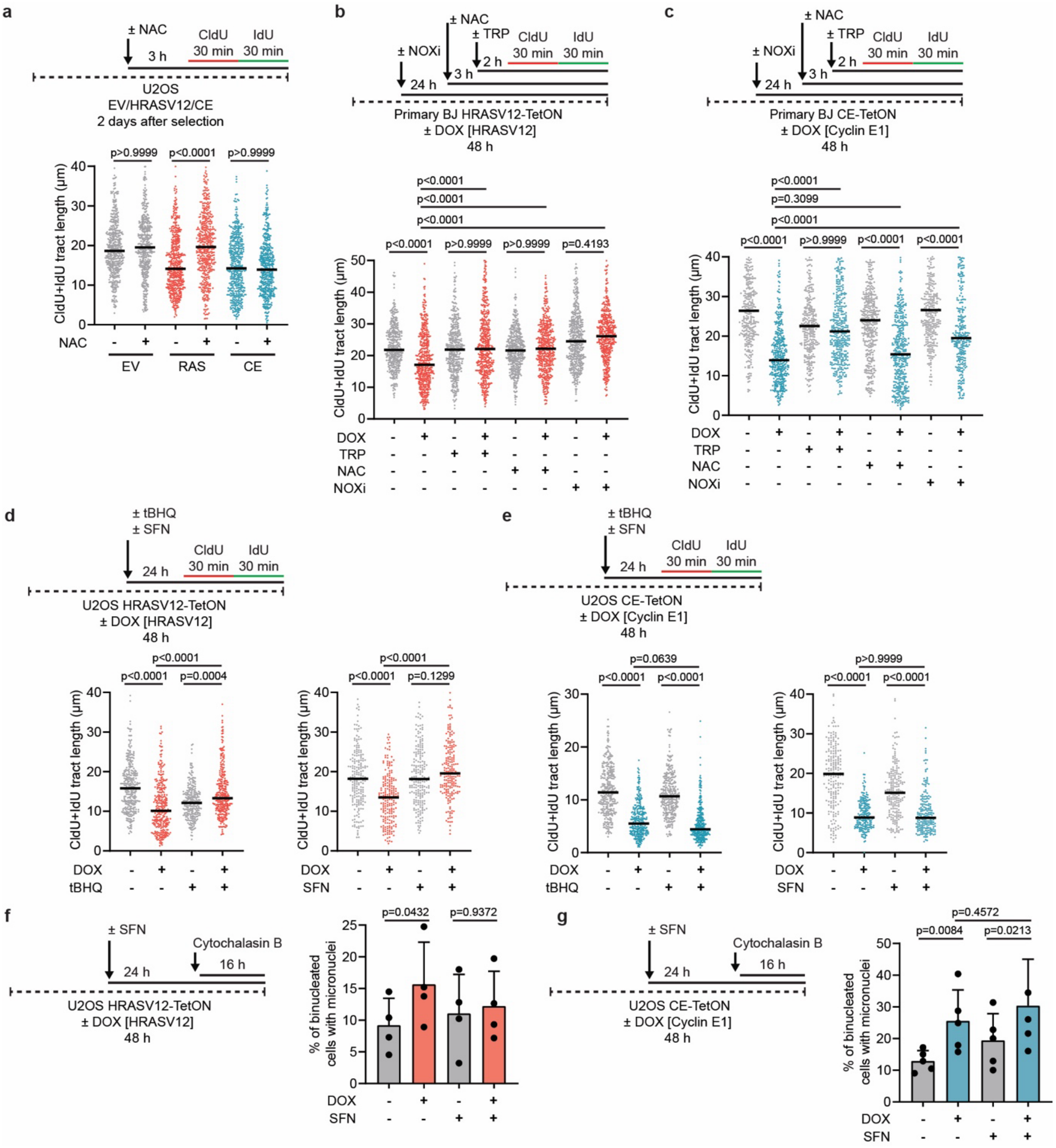

Supplementary Fig 11. HRASV12-induced replication stress is caused by reactive oxygen species generated by NADPH oxidases.

a *Top*: Workflow for DNA fiber labeling in transduced U2OS cells. N-Acetyl-L-cysteine (NAC, 5 mM) was present for 2 h prior to and during the labeling. *Bottom*: Plot of replication tract lengths (CldU+IdU) from three independent experiments (n ≥ 444). EV, empty vector; RAS, HRASV12; CE, cyclin E1. **b** *Top***:** Workflow for DNA fiber labeling in primary BJ HRASV12-TetON fibroblasts. Cells were treated with doxycycline (DOX, 2 μg/ml) for 48 h to induce HRASV12 overexpression. Setanaxib, NADPH oxidase 1/4 inhibitor (NOXi, 1 μM), was present for 24 h prior to and during the labeling. NAC was added as in (**a**). The transcription inhibitor triptolide (TRP, 1 μM) was present for 1 h prior to and during the labeling. *Bottom:* Plot of replication tract lengths (CldU+IdU) from three independent experiments (n ≥ 389). **c** *Top*: Workflow for DNA fiber labeling in primary BJ CE-TetON

fibroblasts. Cells were treated with DOX (2 µg/ml) for 48 h to induce cyclin E1 overexpression. NOXi, NAC and TRP were added as in (**b**). *Bottom:* Plot of values of replication tract lengths (CldU+IdU) from three independent experiments (n ≥ 294). **d** *Top:* Workflow for DNA fiber labeling in U2OS HRASV12-TetON cells. Cells were treated with DOX (1 µg/ml) for 48 h to induce HRASV12 overexpression. Tert-butylhydroquinone (tBHQ, 10 µM) or sulforaphane (SFN, 10 µM) were present for 24 h prior to and during the labeling. *Bottom-left*: Plot of replication tract lengths (CldU+IdU) from two independent experiments with tBHQ (n ≥ 255). *Bottom-right* Plot of replication tract lengths (CldU+IdU) from two independent experiments with SFN (n ≥ 200). **e** *Top:* Workflow for DNA fiber labeling in U2OS CE-TetON cells. Cells were treated with DOX (1 µg/ml) for 48 h to induce cyclin E1 overexpression. tBHQ and SFN were added as in (**d**). *Bottom-left*: Plot of replication tract lengths (CldU+IdU) from two independent experiments with tBHQ (n ≥ 304). *Bottom-right* Plot of replication tract lengths (CldU+IdU) from two independent experiments with SFN (n ≥ 202). **f**, **g** *Left:* Workflow for micronucleus assays with U2OS HRASV12-TetON (**f**) and U2OS CE-TetON (**g**) cells. Cells were treated with DOX (1 µg/ml) for 48 h to induce oncogene overexpression. SFN (10 µM) was added for 24 h. Cytochalasin B (2 µg/ml) was added for the last 16 h to enrich for once-divided binucleated cells. *Right*: Quantification of binucleated cells with micronuclei. **a-e** Black horizontal lines indicate the median; p-values were calculated by Kruskal-Wallis test followed by Dunn's multiple comparisons test. **f**, **g** Data are presented as mean ± SD, n = 4-5; p-values were calculated by one-way one-way ANOVA followed by Tukey's multiple comparisons test. At least 100 binucleated cells were analyzed per experiment for each condition. Source data are provided as a Source Data file.

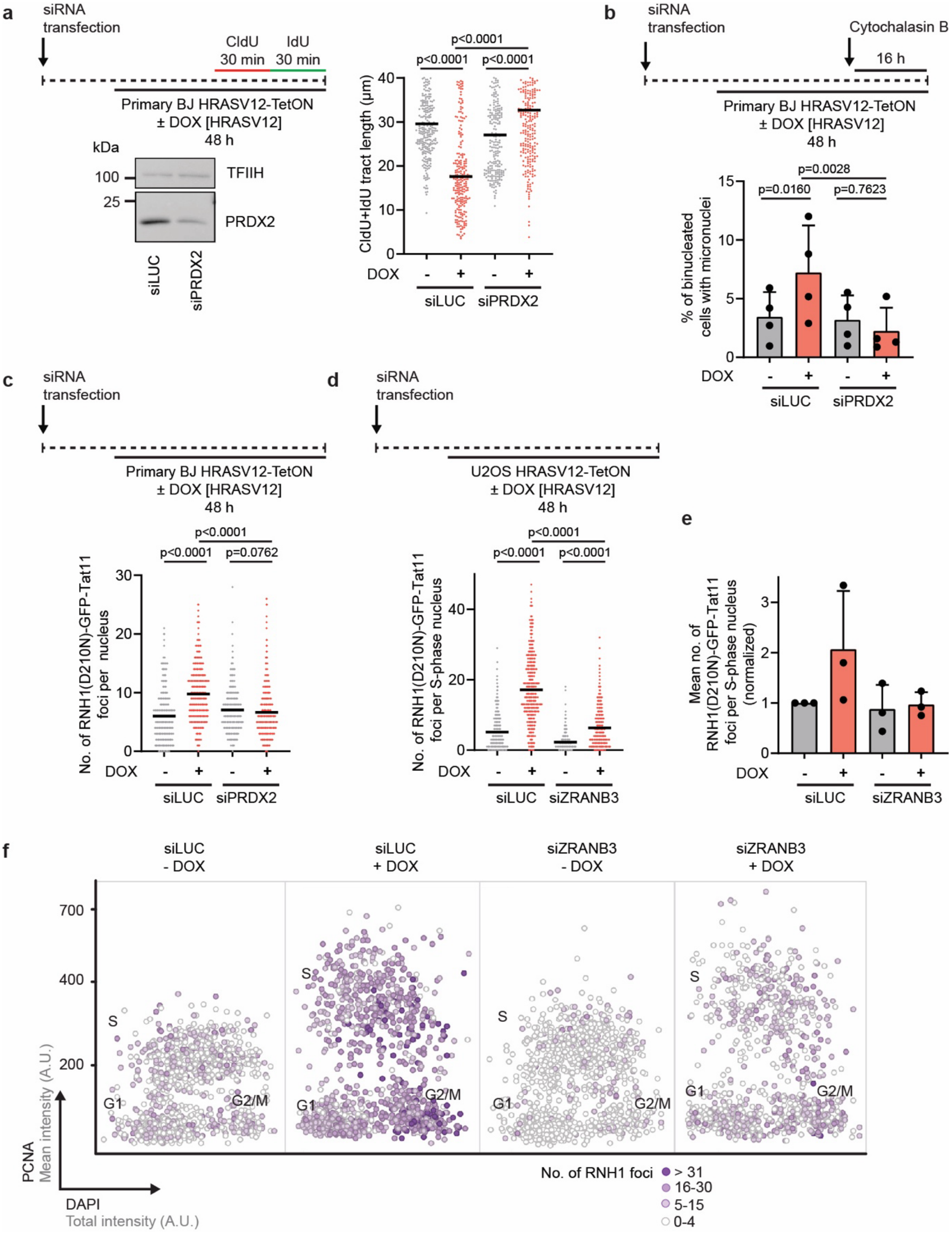

Supplementary Figure 12. HRASV12-induced replication stress depends on the replisome-associated ROS sensor PRDX2.

a *Top-left*: Workflow for DNA fiber labeling in primary BJ HRASV12-TetON fibroblasts transfected with siLUC or siPRDX2. Cells were treated with doxycycline (DOX, 2 µg/ml) for 48 h to induce HRASV12 overexpression. *Bottom-left*: Western blot analysis of PRDX2 expression levels. *Right:* Plot of replication tract lengths (CldU+IdU) from two independent

experiments (n ≥ 248). **b** *Top*: Workflow for micronucleus assays with primary BJ HRASV12-TetON fibroblasts transfected with siLUC or siPRDX2. Cells were treated with DOX (2 µg/ml) for 48 h to induce HRASV12 overexpression. Cytochalasin B (2 µg/ml) was added for the last 16 h to enrich for once-divided binucleated cells. *Bottom*: Quantification of binucleated cells with micronuclei. Data are presented as mean ± SD, n = 4; p-values were calculated by one-way ANOVA followed by Tukey's multiple comparisons test. At least 100 binucleated cells were analyzed per experiment for each condition. **c** *Top*: Workflow for R-loop assays with primary BJ HRASV12-TetON fibroblasts transfected with siLUC or siPRDX2. Cells were treated with DOX (2 µg/ml) for 48 h to induce HRASV12 overexpression. Cells were stained with purified recombinant RNH1(D210N)-GFP-Tat11 protein. *Bottom:* Quantification of the number of RNH1(D210N)-GFP-Tat11 foci per nucleus (n ≥ 309). **d** *Top:* Workflow of R-loop assays with U2OS HRASV12-TetON cells transfected with siLUC or siPRDX2. Cells were treated with DOX (1 µg/ml) for 48 h to induce HRASV12 overexpression. Cells were stained with recombinant RNH1(D210N)-GFP-Tat11 protein and PCNA antibody. PCNA staining was used to identify S-phase cells. *Bottom*: Quantification of the number of RNH1(D210N)-GFP-Tat11 foci per S-phase nucleus (n = 393). A representative plot from three independent experiments yielding similar results is shown. **e** Plot of the mean values of the data sets represented in (**d**). Data are normalized to the siLUC/DOX(-) condition. Error bars represent SD, n = 3. **f** Scatter plot of total DAPI (x-axis) and mean PCNA (y-axis) intensities in individual cells represented in (**d**). Colors indicate the number of RNH1(D210N)-GFP-Tat11 foci, as shown in the legend on the right. For visualisation, 890-1000 cells per condition were randomly selected. Clusters of G1, S and G2 cells are marked in the plots. **a**, **c**, **d** Black horizontal lines indicate the median (**a**) or the mean (**c**, **d**); p-values were calculated by Kruskal-Wallis test followed by Dunn's multiple comparisons test. Source data are provided as a Source Data file.

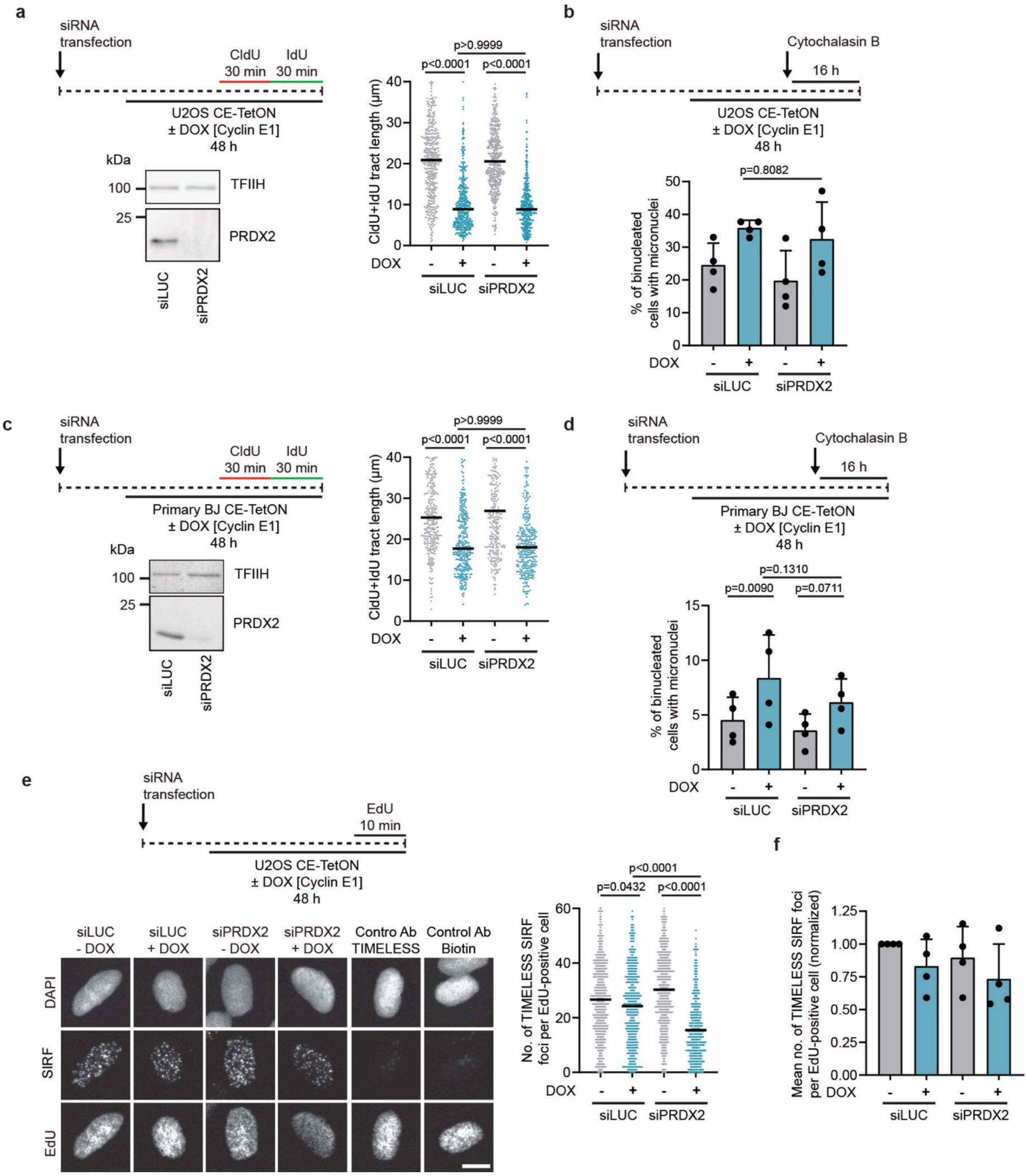

Supplementary Fig. 13. Replication stress induced by cyclin E1 overexpression is independent of PRDX2.

a *Top-left*: Workflow for DNA fiber labeling in U2OS CE-TetON cells transfected with siLUC or siPRDX2. Cells were treated with doxycycline (DOX, 1 µg/ml) for 48 h to induce cyclin E1 overexpression. *Bottom-left*: Western blot analysis of PRDX2 expression levels. *Right:* Plot of replication tract lengths (CldU+IdU) from three independent experiments (n ≥ 419). **b** *Top*: Workflow for micronucleus assays with U2OS CE-TetON cells transfected with siLUC or siPRDX2. Cyclin E1 overexpression was induced as in (**a**). Cytochalasin B (2 µg/ml) was added for the last 16 h to enrich for once-divided binucleated cells. *Bottom*: Quantification of binucleated cells with micronuclei. Data are mean ± SD, n = 4; p-values were calculated by one-way ANOVA followed by Tukey's multiple comparisons test. At least 100 binucleated

cells were analyzed per experiment for each condition. **c** *Top-left*: Workflow for DNA fiber labeling in primary BJ CE-TetON fibroblasts transfected with siLUC or siPRDX2. Cells were treated with DOX (2 µg/ml) for 48 h to induce cyclin E1 overexpression. *Bottom-left*: Western blot analysis of PRDX2 expression levels. *Right:* Plot of replication tract lengths (CldU+IdU) from three independent experiments ($n \geq 305$). **d** *Top*: Workflow for micronucleus assays with primary BJ CE-TetON transfected with siLUC or siPRDX2. Cyclin E1 overexpression was induced as in (c). Cytochalasin B (2 µg/ml) was added for the last 16 h. *Bottom*: Quantification of binucleated cells with micronuclei. Data are mean ± SD, n = 4; p-values were calculated by one-way ANOVA followed by Tukey's multiple comparisons test. At least 100 binucleated cells were analyzed per experiment for each condition. **e** *Top-left*: Workflow for SIRF assays with U2OS CE-TetON transfected with siLUC or siPRDX2. Cells were treated with DOX (1 µg/ml) for 48 h to induce cyclin E1 overexpression. For the last 10 min, cells were treated with EdU (25 µM), and EdU signal was used to identify S-phase cells. *Bottom-left:* Representative images of DAPI, SIRF, and EdU channels. Images for control conditions with only anti-TIMELESS or only anti-biotin antibody are also shown. Scale bar, 10 µm. *Right:* Quantification of the number of TIMELESS SIRF foci per S-phase nucleus (n = 507). A representative plot from four independent experiments yielding similar results is shown. **f** Plot of the mean values of the data sets represented in (**e**). Data are normalized to the siLUC/DOX(-) condition. Error bars represent SD, n = 3. **a**, **c**, **e** Black horizontal lines indicate the median (**a**, **c**) or the mean (**e**); p-values were calculated by Kruskal-Wallis test followed by Dunn's multiple comparisons test. **b**, **d** Data are presented as mean ± SD, n = 4; p-values were calculated by one-way ANOVA followed by Tukey's multiple comparisons test. Source data are provided as a Source Data file.

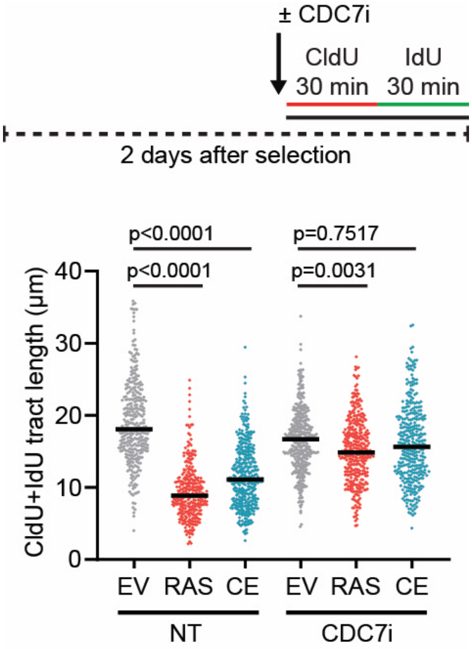

Supplementary Fig. 14. CDC7 inhibition rescues HRASV12- and cyclin E1-induced replication fork slowing in U2OS cells.

Top: Workflow for DNA fiber labeling in transduced U2OS cells two days after selection. Where required, CDC7 inhibitor XL413 (CDC7i, 2 µM) was present during the labeling. *Bottom*: Plot of values of replication tract lengths (CldU+IdU) from three independent experiments (n ≥318). Black horizontal lines indicate the median; p-values were calculated by Kruskal-Wallis test followed by Dunn's multiple comparisons test. EV, empty vector; RAS, HRASV12; CE, cyclin E1. Source data are provided as a Source Data file.
