# Supplementary material for: Distinct mechanisms of replication stress induced by oncogenic RAS and cyclin E1 converge on R-loop-dependent fork reversal
Source: Nat Commun. 2026 Apr 3;17:4784. doi: 10.1038/s41467-026-71353-8 (PMC13219726; doi:10.1038/s41467-026-71353-8)
Supplement: Supplementary file 2 — Reporting Summary [file 41467_2026_71353_MOESM2_ESM.pdf]

nature portfolio

Corresponding author(s): Pavel Janscak

Last updated by author(s): Mar 5, 2026

Reporting Summary

Nature Portfolio wishes to improve the reproducibility of the work that we publish. This form provides structure for consistency and transparency in reporting. For further information on Nature Portfolio policies, see our Editorial Policies and the Editorial Policy Checklist.

Statistics

For all statistical analyses, confirm that the following items are present in the figure legend, table legend, main text, or Methods section.

n/a	Confirmed	
☐	☒	The exact sample size (*n*) for each experimental group/condition, given as a discrete number and unit of measurement
☐	☒	A statement on whether measurements were taken from distinct samples or whether the same sample was measured repeatedly
☐	☒	The statistical test(s) used AND whether they are one- or two-sided<br>*Only common tests should be described solely by name; describe more complex techniques in the Methods section.*
☒	☐	A description of all covariates tested
☐	☒	A description of any assumptions or corrections, such as tests of normality and adjustment for multiple comparisons
☐	☒	A full description of the statistical parameters including central tendency (e.g. means) or other basic estimates (e.g. regression coefficient) AND variation (e.g. standard deviation) or associated estimates of uncertainty (e.g. confidence intervals)
☐	☒	For null hypothesis testing, the test statistic (e.g. *F*, *t*, *r*) with confidence intervals, effect sizes, degrees of freedom and *P* value noted<br>*Give P values as exact values whenever suitable.*
☒	☐	For Bayesian analysis, information on the choice of priors and Markov chain Monte Carlo settings
☒	☐	For hierarchical and complex designs, identification of the appropriate level for tests and full reporting of outcomes
☒	☐	Estimates of effect sizes (e.g. Cohen's *d*, Pearson's *r*), indicating how they were calculated

Our web collection on statistics for biologists contains articles on many of the points above.

Software and code

Policy information about availability of computer code

Data collection	QIBC data collection was performed using Olympus ScanR Acquisition software (version 3.5.0); electron microscopy images were acquired by SerialEM (version 3.8.6), other microscopy images were acquired by Leica Application Suite X 3.6.0.20104.
Data analysis	QIBC microscopy images: GFP foci, γH2AX foci, 53BP1 foci, PCNA:RNAPII PLA foci, PCNA foci and TIMELESS SIRF foci were analyzed by Olympus ScanR Analysis software (version 3.5.0); DNA fibers, anaphase bridges, slot blot and micronuclei were analyzed by ImageJ software (version 1.54F); data analysis was done in GraphPad Prism 8 software (version 8.4.3) and TIBCO Spotfire software (version 10.10.1). Electron microscopy images were analyzed by SerialEM (version 3.8.6) and ImageJ software (version 1.54F).

For manuscripts utilizing custom algorithms or software that are central to the research but not yet described in published literature, software must be made available to editors and reviewers. We strongly encourage code deposition in a community repository (e.g. GitHub). See the Nature Portfolio guidelines for submitting code & software for further information.

Data

Policy information about availability of data

All manuscripts must include a data availability statement. This statement should provide the following information, where applicable:

- Accession codes, unique identifiers, or web links for publicly available datasets
- A description of any restrictions on data availability
- For clinical datasets or third party data, please ensure that the statement adheres to our policy

The authors declare that all data supporting the findings of this study are provided in the paper and its Supplementary information. The source data for Figs 1-8 and Supplementary Figs. 1-14 are provided as a Source Data file. Original microscopy images are too numerous and large to be uploaded to a public repository but can be made available upon request.

Research involving human participants, their data, or biological material

Policy information about studies with human participants or human data. See also policy information about sex, gender (identity/presentation), and sexual orientation and race, ethnicity and racism.

Reporting on sex and gender	N/A
Reporting on race, ethnicity, or other socially relevant groupings	N/A
Population characteristics	N/A
Recruitment	N/A
Ethics oversight	N/A

Note that full information on the approval of the study protocol must also be provided in the manuscript.

Field-specific reporting

Please select the one below that is the best fit for your research. If you are not sure, read the appropriate sections before making your selection.

☒ Life sciences ☐ Behavioural & social sciences ☐ Ecological, evolutionary & environmental sciences

For a reference copy of the document with all sections, see nature.com/documents/nr-reporting-summary-flat.pdf

Life sciences study design

All studies must disclose on these points even when the disclosure is negative.

Sample size	Sample size for all experiments shown [n>90 in 2 or more independent experiments for replication tract lengths; n>30 in 3 or more independent experiments for sister fork asymmetry; n>100 in 3 or more independent experiments for micronuclei; n>25 in 3 or more independent experiments for anaphase bridges; n>393, usually n>600, in 2 or more independent experiments for QIBC (the only exception is Supplementary Fig. 12c, where n>309 was obtained from a single experiment; this result is supported by data from another cell line); and n>40 in 3 independent experiments using electron microscopy] was chosen to obtain statistical power, in conformity to accepted standard sample size in a number of previous publications using these approaches:<br><br>Berti et al., Nat Commun., DOI: 10.1038/s41467-020-17324-z<br>Mijic et al., Nat Commun., DOI: 10.1038/s41467-017-01164-5<br>Vujanovic et al., Mol Cell, DOI: 10.1016/j.molcel.2017.08.010<br>Mutreja et al., Cell Rep., DOI: 10.1016/j.celrep.2018.08.019<br>Andrs et al., Nat Commun., DOI:10.1038/s41467-023-37341-y
Data exclusions	No data were excluded from any of the analyses.
Replication	For all experiments, the number of biological replicates is indicated. With the only exception of Supplementary Fig. 12c, all data were reproduced two or more times, and representative data are shown in the figures.
Randomization	We were working with asynchronously cycling cell populations or individual replicating DNA molecules from these cell populations, hence further randomization was not necessary for our approaches. In the cell cycle graph, 750 or 1000 cells per condition were randomly selected for visualization purposes. For each analyzed cell, a random number was generated using the RAND() function in Excel, and the cells were then ordered according to the newly generated numbers. The first 1000 or 750 cells in each condition were used to create the cell cycle graph.
Blinding	Individual repetitions for DNA fiber analysis, micronuclei analysis, anaphase bridges analysis and Electron Microscopy were blinded to the

Blinding	investigators.<br>For the automated QIBC screen blinding was not necessary due to its intrinsically unbiased nature.

Reporting for specific materials, systems and methods

We require information from authors about some types of materials, experimental systems and methods used in many studies. Here, indicate whether each material, system or method listed is relevant to your study. If you are not sure if a list item applies to your research, read the appropriate section before selecting a response.

Materials & experimental systems

n/a	Involved in the study
☐	☒ Antibodies
☐	☒ Eukaryotic cell lines
☒	☐ Palaeontology and archaeology
☒	☐ Animals and other organisms
☒	☐ Clinical data
☒	☐ Dual use research of concern
☒	☐ Plants

Methods

n/a	Involved in the study
☒	☐ ChIP-seq
☒	☐ Flow cytometry
☒	☐ MRI-based neuroimaging

Antibodies

Antibodies used	The primary antibodies:<br>HRAS (259) rat monoclonal (sc-35, Santa Cruz Biotechnology); HRAS (C-20) rabbit polyclonal (sc-520, Santa Cruz Biotechnology); HRAS rabbit polyclonal (GTX 116041, GeneTex); Cyclin E (HE12) mouse monoclonal (sc-247, Santa Cruz Biotechnology); TFIIH p89 (S-19) rabbit polyclonal (sc-293, Santa Cruz Biotechnology); RNASE H1 (A-9) mouse monoclonal (sc-365783, Santa Cruz Biotechnology); ZRANB3 rabbit polyclonal (23111- 1-AP, Proteintech); SMARCAL1 (D3P5I) rabbit monoclonal (44717S, Cell Signaling Technology); GAPDH (D16H11) rabbit monoclonal (#5174, Cell Signaling Technology); GAPDH (0411) mouse monoclonal (sc-47724, Santa Cruz), β-Tubulin (TUB 2.1) mouse monoclonal (T4026, Sigma-Aldrich); MUS81 (B-12) mouse monoclonal (sc-376661, Santa Cruz Biotechnology); MUS81 (MTA30 2G10/3) mouse monoclonal (sc-53382, Santa Cruz Biotechnology); PRIMPOL rat polyclonal (a gift from Juan Mendez); RECQ1 rabbit polyclonal (NB100-618, Novus Biological); LIG4 (D-8) mouse monoclonal (sc-271299, Santa Cruz Biotechnology), ELL (B-4) mouse monoclonal (sc-398959, Santa Cruz Biotechnology), PRDX2 (A-2) mouse monoclonal (sc-515428, Santa Cruz), ssDNA antibody (MAB3868, Millipore) and S9.6 antibody (ENH001, Kerafast); PCNA rabbit polyclonal antibody (ab18197, Abcam); PCNA rat polyclonal (ab252848, Abcam); Cyclin A mouse monoclonal (sc-271682, Santa Cruz Biotechnology); 53BP1 (H-300) rabbit polyclonal (sc22760, Santa Cruz); γH2AX mouse monoclonal (Ser139) (05-636, Millipore); RNA polymerase II RPB1 mouse monoclonal (920204, Biolegend); biotin mouse monoclonal (200-002-211, Jackson ImmunoResearch); Timeless rabbit monoclonal (ab109512, Abcam); BrdU (CldU) rat (ab6326, Abcam) and BrdU (IdU) mouse (347580, BD Biosciences).<br>The secondary antibodies:<br>goat anti-rabbit IgG-HRP (A0545, Sigma-Aldrich); goat anti-mouse IgG-HRP (A4416, Sigma-Aldrich), goat anti-rat IgG-HRP (A9037, Sigma-Aldrich), Alexa Fluor 488 Goat Anti-Mouse IgG (A11001, Thermo Fisher Scientific), Alexa Fluor 647 Goat Anti-Rabbit IgG (A-21245, Thermo Fisher Scientific), Alexa Fluor 555 Goat Anti-Rat (A21434, Thermo Fisher Scientific), donkey anti-rat Cy3 (712-166-153, Jackson ImmunoResearch).
Validation	All antibodies, except for PRIMPOL antibody, were purchased from commercial vendors and were extensively validated regarding species specificity, cross reactivity with other antigens, and their use in various applications. This information is readily available on the supplier websites. In addition, the antibodies directed against the ZRANB3, SMARCAL1, MUS81, LIG4, RECQ1, ELL, PRIMPOL and PRDX2 were further validated for our approaches using siRNA directed against the protein of interest and/ or the corresponding knock-out cell line.

Eukaryotic cell lines

Policy information about cell lines and Sex and Gender in Research

Cell line source(s)	Cell line:<br>BJ-hTert HRASV12ER-TAM was prepared in Agami and de Vita labs (doi: 10.1210/me.2004-0172)<br>RPE1-cE (RPE1-pRetroX-Tet-On Advanced cells stably transfected with pRetroX-Tight-Pur-CCNE1) was prepared in Marcel A. T. M. van Vugt lab (doi: 10.1038/s41389-020-00270-2)<br>U2OS ZRANB3-WT was prepared in Massimo Lopes lab (doi: 10.1016/j.molcel.2017.08.010)<br>U2OS ZRANB3-KO was prepared in David Cortez lab (doi: 10.1016/j.molcel.2017.08.010)<br>U2OS (ATCC; HTB-96)<br>PhoenixAMPHO (ATCC; CRL-3213)<br>HEK293T (ATCC; CRL-3216)<br>HeLa Kyoto (Cancer Research UK; CVCL_1922)<br>HeLa Kyoto MUS81 knockout was prepared in Joao Matos lab (doi: 10.1016/j.molcel.2019.10.026)<br>U2OS T-REx RNH1-GFP was prepared in Janscak lab (doi: 10.1016/j.molcel.2019.10.026)<br>U2OS T-REx RNH1-D210N-GFP was prepared in Janscak lab (doi: 10.1016/j.molcel.2018.11.036)<br>Primary BJ-HRASV12 TetON was obtained from Prof. Jiri Bartek (doi: 10.1016/j.molonc.2014.11.001)<br>Primary BJ-CE TetON was obtained from Prof. Jiri Bartek (doi: 10.1016/j.molonc.2014.11.001)

	U2OS pLVX-TetOne-Puro-HRASV12 was prepared in Janscak lab and was described in this study.<br>U2OS pLVX-TetOne-Puro-CCNE1 was prepared in Janscak lab and was described in this study
Authentication	None of the cell lines were authenticated in house for this manuscript.
Mycoplasma contamination	We routinely do mycoplasma testing on our cell lines.
Commonly misidentified lines<br>(See ICLAC register)	No commonly misidentified lines were used in this study.

Plants

Seed stocks	N/A
Novel plant genotypes	N/A
Authentication	N/A
